# Antiretroviral treatment-induced dyslipidemia in HIV-infected patients is influenced by the *APOC3*-related rs10892151 polymorphism

**DOI:** 10.1186/1471-2350-12-120

**Published:** 2011-09-22

**Authors:** Gerard Aragonès, Carlos Alonso-Villaverde, Pedro Pardo-Reche, Anna Rull, Raúl Beltrán-Debón, Esther Rodríguez-Gallego, Laura Fernández-Sender, Jordi Camps, Jorge Joven

**Affiliations:** 1 Centre de Recerca Biomèdica, Universitat Rovira i Virgili, Institut d'Investigació Sanitària Pere Virgili (IISPV), Reus, Spain; 2 Servei de Medicina Interna, Hospital Son Llàtzer, Palma, Illes Balears, Spain; 3 Servei de Medicina Interna, Hospital Universitari de Sant Joan, Institut d'Investigació Sanitària Pere Virgili (IISPV), Reus, Spain

## Abstract

**Background:**

The recently observed association between the APOC3-related rs10892151 polymorphism and serum triglyceride levels has prompted us the possibility to explore whether this genetic variant may play a major role in human immunodeficiency virus (HIV)/antiretroviral therapy-induced dyslipidemia.

**Methods:**

We determined the rs10892151 genotype distribution and serum apolipoprotein (apo) C-III concentration in a group of HIV-infected patients (*n *= 208) and in a group of age and sex-matched healthy volunteers (*n *= 200). Circulating lipid and lipoprotein levels were followed for 12 months after antiretroviral treatment initiation in the HIV-infected group.

**Results:**

There were no significant variations in the frequency of the A allele between the healthy and HIV-infected groups (7.5 vs. 8.6%, respectively; p = 0.7); additionally, the A allele was not related to serum apo C-III concentration. However, among patients receiving protease inhibitor (PI) treatment, carriers of the A allele had significantly increased serum triglyceride (5.76 ± 2.54 mmol/L) and total cholesterol (6.63 ± 2.85 mmol/L) concentrations together with depressed levels of HDL-cholesterol (0.75 ± 0.3 mmol/L) when compared with patients not carrying the allele (2.43 ± 1.32, 5.2 ± 2.17 and 1.24 ± 0.4 mmol/L, respectively) at the end of the study. This effect was only evident for HDL-cholesterol concentration when patients were treated with non-nucleoside reverse transcriptase inhibitors (1.05 ± 0.4 vs. 1.28 ± 0.4 mmol/L).

**Conclusions:**

The A allelic variant of the rs10892151 polymorphism is not associated with serum apo C-III concentration, but predisposes HIV-infected patients to less favorable lipid profile, particularly in those patients treated with PIs.

## Background

The introduction of antiretroviral therapies has led to a remarkable increase in the life expectancy of patients with human immunodeficiency virus (HIV) infection. Unfortunately, current treatment may cause a wide spectrum of metabolic disturbances and comorbid conditions, with cardiovascular disease as an important example [1]. Dyslipidemia is particularly frequent and is mostly characterized by hypertriglyceridemia and low HDL-cholesterol concentrations. Although this phenomenon has been attributed, at least in part, to the use of protease inhibitors (i.e., ritonavir or ritonavir-boosted treatment), dyslipidemia is also observed in treatment-*naïve *HIV-infected patients, suggesting that HIV infection itself has a metabolically deleterious effect [2,3].

Susceptibility to developing these metabolic derangements, specifically hypertriglyceridemia, varies among individuals and could be influenced by genetic variability [4]. In an attempt to find new candidate genes responsible for variations in lipid concentrations, we have explored the rs10892151 polymorphism, which is located within an intron of the *DSCAML1 *(Down syndrome cell adhesion molecule like-1) gene and is in linkage disequilibrium with a loss-of-function mutation in the *APOC3 *gene. Carriers of this null mutation have low circulating apolipoprotein (apo) C-III levels and reduced fasting and post-prandial triglyceride concentrations [5], which is likely due to the well-established function of apo C-III as an inhibitor of lipoprotein lipase [6]. Other functions attributed to apo C-III include a reduction in the hepatic uptake of apo B-containing lipoproteins [7,8] as well as an increase in the catabolism of high-density lipoprotein (HDL) particles [9], adhesion of monocytes to vascular endothelial cells [10], and the activation of inflammatory signaling pathways [11].

Because HIV-associated dyslipidemia is accompanied by increases in apoB-containing lipoproteins, impaired lipolysis, and decreases in HDL-cholesterol levels [12], we hypothesized that in HIV-infected patients, the rs10892151 polymorphism could be an important genetic factor influencing the deleterious effect of HIV on lipid profile. Hence, we investigated treatment-induced lipid and lipoprotein changes in HIV-infected patients who were segregated with respect to their treatment strategy and their rs10892151 genotype.

## Methods

### Subjects and study design

We recruited patients from among the participants of a longitudinal project assessing atherosclerosis in HIV-infected patients [13,14] from January 2001 through December 2004; all subjects agreed to participate in the present study and provided informed consent. The initial time-point of this study was the commencement of therapy with protease inhibitors (PIs) or with non-nucleoside reverse transcriptase inhibitors (NNRTIs) based schemes. The antiretroviral adjuvant drugs were zidovudine, lamivudine or stavudine. Follow-up was conducted at 3, 6, and 12 months after treatment initiation. Candidates for inclusion were *naïve *patients, and patients previously exposed to antiretroviral treatment who had discontinued treatment for at least 6 months. No patient was being treated with lipid-lowering drugs at the commencement of the study, but 20 patients were treated with fluvastatin (80 mg/day) during the follow-up. For the purpose of this study, only patients who remained on the same antiretroviral treatment regimen during follow-up were included in the analysis. For the control group, we selected 200 age and sex-matched healthy volunteers who participated in an epidemiological study, the details of which have been previously reported [15]. The study was approved by the Ethics Committee of the Hospital Universitari de Sant Joan de Reus.

### Clinical and Laboratory Measurements

Detailed clinical characteristics of each subject were recorded, and a thorough physical examination was performed during the interview. We recorded data regarding HIV infection, including opportunistic infections, mode of HIV transmission, CD4+ T cell count, HIV viral load, presence of lipodystrophy and HCV co-infection status. Body mass index (BMI) was defined as weight (kg)/height^2 ^(m^2^). Fasting serum glucose, cholesterol, triglycerides, highly sensitive C-reactive protein (CRP) and apo C-III concentrations were measured with the LXi 725-Synchron (Beckman Coulter, Fullerton, California, USA) automatic analyzer using enzymatic assays or chemiluminescent immunoassays. HDL- and LDL-cholesterol levels were measured as described [16,17]. Serum concentration of monocyte chemoattractant protein-1 (MCP-1) was measured with an enzyme-linked immunosorbent assay (Human MCP-1 ELISA, PeproTech, London, UK).

### Genotyping

Participants were genotyped for the rs10892151 polymorphism using the TaqMan 5' allelic discrimination assay by the custom TaqMan SNP Genotyping Assay *C_3239453_10 *(Applied Biosystems, Foster City, CA) [18]. Amplifications were performed in a 7900HT Sequencing Detection System (Applied Biosystems) for continuous fluorescence monitoring.

#### Statistical analyses

Analysis was performed using SPSS, version 18.0 (SPSS Inc., Chicago, IL). All data are presented as mean ± SD, except where otherwise stated. The Kolmogorov-Smirnov test was used to check whether data were normally distributed. The Hardy-Weinberg equilibrium (HWE) for the SNP was tested with the χ^2 ^test. Allele and genotype frequencies between cases and controls were compared with the χ^2 ^analysis. Differences in baseline characteristics between the two treatment groups were assessed through *the χ*^2 ^and Mann-Whitney *U *test for categorical and continuous variables, respectively. To asses whether changes in lipid levels at months 0, 3, 6, and 12 were significantly different from baseline in each treatment group, and whether those changes were significantly different between the two treatment groups, we used linear mixed models including the interaction between treatment group and month of therapy as categorical. Linear mixed models allowed us to accommodate multiple measures per person. Univariate and multivariate analyses were performed, with adjustment for confounding factors such as sex, age, BMI, co-infection with HCV, lipodystrophy and lipid-lowering therapy.

## Results

### Characteristics of participants

Of 208 Caucasian subjects who met selection criteria, 113 (54.3%) initiated NNRTI and 95 (45.7%) PI based scheme. The second component of the regimen were two non nucleoside reverse transcriptase inhibitor [zidovudine (70.2%), lamivudine (91.3%) or stavudine (38.5%) in patients treated with NNRTI, and zidovudine (77.8%), lamivudine (84.8%) or stavudine (37.4%) in patients treated with PI; p = 0.8]. Of the 208 patients initially selected, 201, 198 and 192 of them remained on the same ART regimen at months 3, 6 and 12 of follow-up, respectively. 160 (76.9%) were treatment-*naïve *patients (95 in NNRTI group and 65 in the PI group, p = 0.62), and 48 (23.1%) were patients reinitiating antiretroviral therapy after treatment interruption for at least 6 months (18 in NNRTI group and 30 in the PI group, p = 0.79).

The baseline clinically relevant characteristics of the patients included in the study are summarized in Table 1. The mean time from diagnosis was 7.24 ± 0.36 years, and 133 (63.9%) patients were co-infected with hepatitis C virus. These patients were either current or past intravenous drug users (60.1%) or became infected as a result of sexual intercourse. The baseline examination revealed that most patients were heavy smokers, were relatively young, were not significantly obese and had normal blood pressure values.

**Table 1:** Baseline main clinical and demographic characteristics of HIV-infected patients based on rs10892151 genotype

Characteristics at study entry	G/G carriers (*n *= 190)	A/G carriers (*n *= 18)
Age, years	39.1 (6.9)	37.9 (7.0)
Gender, n (%)		
Male	127 (66.8)	13 (72.2)
Female	63 (33.2)	5 (27.8)
BMI, kg/m^2^	22.74 (2.9)	23.23 (3.6)
Risk factors for HIV, n (%)		
Intravenous drug use	114 (60.0)	11 (61.1)
Heterosexual contact	57 (30.0)	5 (27.7)
Male homosexual contact	19 (10.0)	2 (11.2)
HCV co-infection, n (%)	115 (60.5)	13 (72.2)
HIV-1 RNA viral load, copies/mL	246582 (776921)	336652 (863524)
CD4+ T cell count, cells/μL	448.9 (285.9)	435.1 (290.9)
Treatment scheme, n (%)		
NNRTI based	103 (54.2)	10 (55.5)
PI based	87 (45.8)	8 (44.5)
Apo C-III, μg/mL	11.27 (4.9)	10.42 (4.7)
Total cholesterol, mmol/L	4.89 (1.2)	4.80 (1.2)
HDL-cholesterol, mmol/L	1.06 (0.4)	1.08 (0.4)
VLDL-cholesterol, mmol/L	0.87 (0.3)	0.64 (0.2)
LDL-cholesterol, mmol/L	2.91 (0.9)	2.88 (0.9)
Triglycerides, mmol/L	2.48 (2.0)	2.40 (2.0)
Glucose, mmol/L	5.39 (0.9)	5.51 (1.0)
CRP, mg/L	4.13 (5.4)	4.91 (6.2)
MCP-1, pg/mL	74.72 (41.8)	81.17 (43.1)

Values are the mean and the standard deviation (SD), unless otherwise indicated. BMI, body-mass index

### rs10892151 genotype distribution

The genotype and allelic frequencies of the rs10892151 polymorphism did not show significant variations between healthy and HIV-infected participants (p = 0.7). A total of 18 subjects (8.6%) from the HIV-infected group and 15 (7.5%) from the healthy group were carriers of the A allele, while 190 (91.4%) HIV-infected patients and 185 (92.5%) healthy subjects were carriers of two G alleles (the wild-type genotype). No subjects were homozygous for the A allele. The A allelic frequency for these populations was similar to that reported in the *National Center for Biotechnology Information SNP public database*; it was not significantly different to that predicted by the Hardy-Weinberg distribution.

When we examined serum apo C-III concentration based on genotypes, no statistically significant differences were observed in serum apo C-III levels among healthy participants [9.21 (3.7) μg/mL in G/G carriers versus 8.91 (3.5) μg/mL in A/G carriers; p = 0.8] or among HIV-infected patients [10.49 (4.8) μg/mL in G/G carriers versus 10.34 (4.8) μg/mL in A/G carriers; p = 0.7]. We also failed to observe significant associations between the duration and nature of antiretroviral treatment or viral load and serum apo C-III concentration as well as between lipid profile and rs10892151 genotype distribution in healthy participants.

### Effects of rs10892151 polymorphism on metabolic variables

Table 2 depicts the main baseline characteristics of HIV-infected patients segregated according to rs10892151 genotype variants and treatment scheme. No significant differences were observed between groups for the variables considered here. It was only in the subset of HIV-infected patients without HCV co-infection that carriers of the A allele displayed a trend towards a favorable lipid profile when compared to non-carriers; however, this difference did not reach statistical significance (data not shown).

**Table 2:** Baseline main metabolic characteristics of HIV-infected patients based on rs10892151 genotype and treatment strategy

	PI-treated patients (*n *= 95)		NNRTI-treated patients (*n *= 113)	
	
	G/G carriers (*n *= 87)	A/G carriers (*n *= 8)	G/G carriers (*n *= 103)	A/G carriers (*n *= 10)
**BMI, kg/m^2^**	22.9 (3.1)	23.4 (3.2)	22.6 (2.9)	23.1 (3.1)
**ApoC-III, μg/mL**	11.12 (5.1)	10.62 (4.6)	11.39 (4.9)	10.26 (4.8)
**Total cholesterol, mmol/L**	4.91 (1.3)	5.06 (1.3)	4.87 (1.2)	4.60 (1.2)
HDL-cholesterol, mmol/L	1.02 (0.4)	1.06 (0.4)	1.09 (0.4)	1.1 (0.4)
**VLDL-cholesterol, mmol/L**	0.82 (0.2)	0.54 (0.1)	0.91 (0.4)	0.72 (0.3)
**LDL-cholesterol, mmol/L**	2.82 (0.8)	2.87 (0.9)	2.88 (0.9)	2.95 (0.9)
**Triglycerides, mmol/L**	2.65 (2.1)	2.78 (2.2)	2.34 (2.2)	2.10 (2.1)
**Glucose, mmol/L**	5.68 (1.0)	5.95 (0.9)	5.15 (0.9)	5.16 (0.9)
**CRP, mg/L**	4.6 (5.4)	5.1 (5.6)	3.73 (5.5)	4.76 (5.7)
**MCP-1, pg/mL**	73.31 (40.3)	78.76 (43.8)	75.92 (42.1)	83.10 (44.8)

Values are the mean and the standard deviation (SD), unless otherwise indicated. BMI, body-mass index; PI, protease inhibitor; NNRTI, non-nucleoside reverse transcritase inhibitor

### Course of lipid disturbances in patients receiving either PI or NNRTI therapy

**Figure 1:**
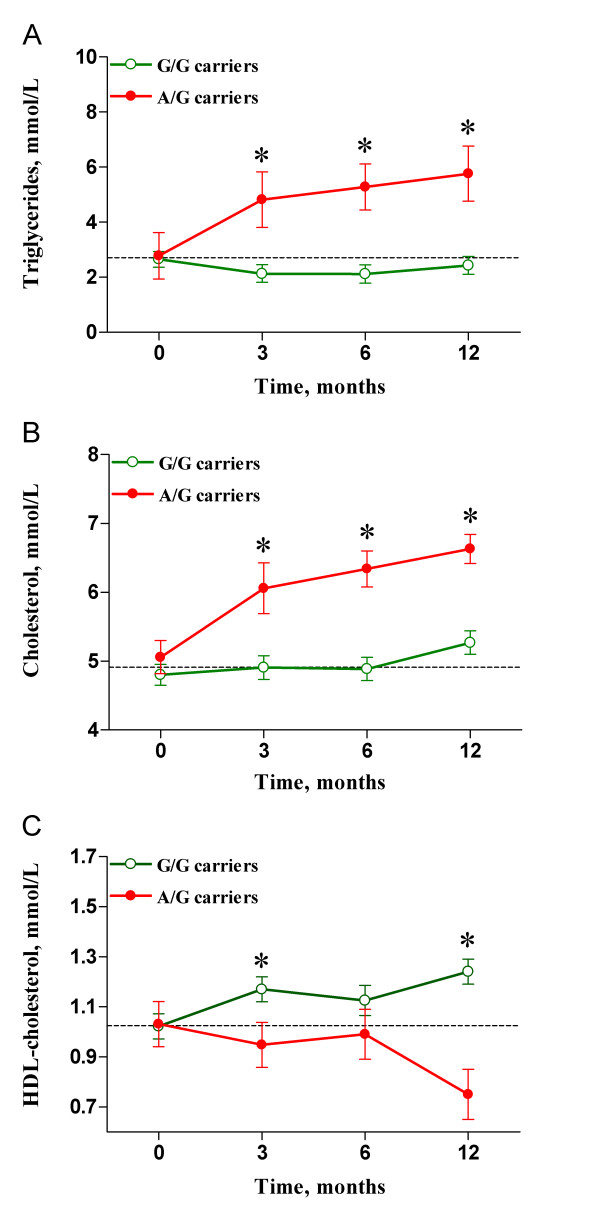
**Lipid profile changes in patients receiving PI therapy**. Carriers of the A allele (closed circles) had significantly increased serum triglyceride (A) and total cholesterol (B) concentrations together with depressed levels of HDL-cholesterol (C) when compared with non-carriers (open circles) at 3, 6, and 12 months after treatment initiation. * indicates p < 0.05.

Because hyperlipidemia is strongly associated with the PI treatment regimen, we focused on a subgroup of 95 patients receiving PIs as a component of their antiretroviral therapy, and we analyzed lipid profile changes over a 12-month follow-up period. As indicated, baseline values of metabolic parameters were comparable based on genotype. However, carriers of the A allele had consistently higher serum triglyceride concentration than non-carriers at three (4.82 ± 2.1 vs. 2.13 ± 1.1 mmol/L; p = 0.001), six (5.28 ± 2.84 vs. 2.21 ± 1.33 mmol/L; p = 0.003), and twelve (5.76 ± 2.54 vs. 2.43 ± 1.32 mmol/L; p = 0.019) months, after adjustment of the data for age, sex, BMI, and HCV co-infection status (Figure 1A). Results were similar for total cholesterol concentration (Figure 1B). Carriers of the A allele presented with higher serum cholesterol values than non-carriers at three (6.12 ± 1.5 vs. 4.9 ± 1.3 mmol/L; p = 0.01), six (6.39 ± 1.5 vs. 4.91 ± 1.1 mmol/L; p = 0.006), and twelve (6.63 ± 1.9 vs. 5.2 ± 1.4 mmol/L; p = 0.034) months. Also, carriers of the A allele had lower HDL-cholesterol levels than non-carriers at three (0.94 ± 0.4 vs. 1.17 ± 0.4 mmol/L; p = 0.016), six (1.01 ± 0.3 vs. 1.07 ± 0.4 mmol/L; not significant), and twelve (1.24 ± 0.5 vs. 0.75 ± 0.2 mmol/L; p = 0.05) months after adjustment the data for age, sex, BMI, HCV co-infection status and triglyceride levels (Figure 1C). Both, LDL and VLDL-cholesterol concentrations showed a tendency toward increase in carriers of the A allele, but these differences did not reach statistical significance (data not shown).

**Figure 2:**
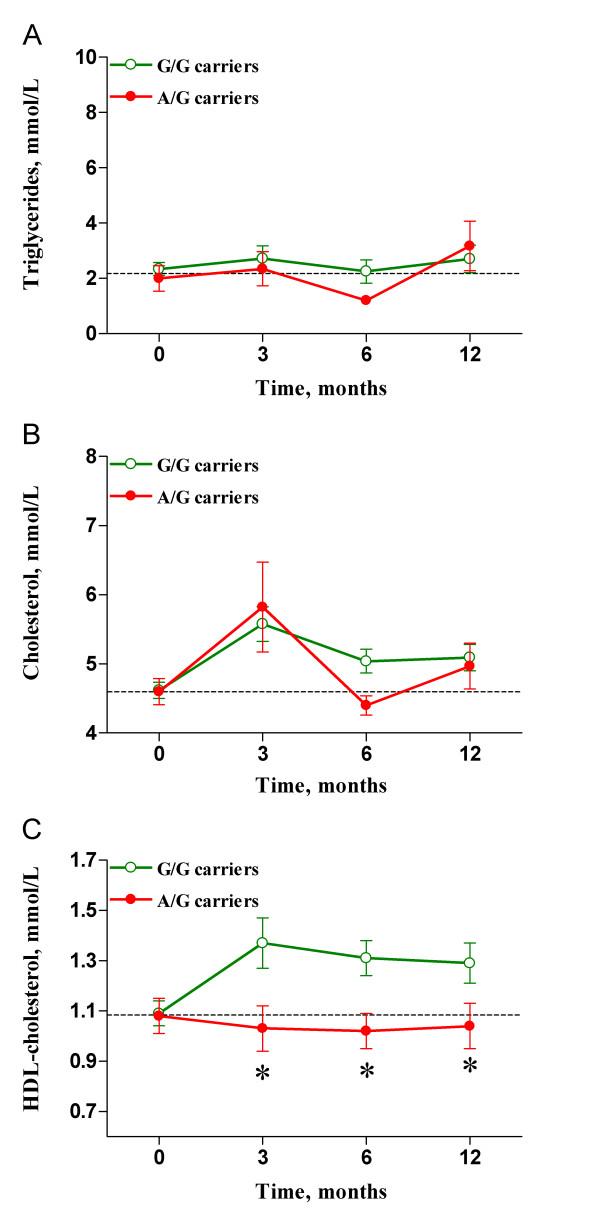
**Lipid profile changes in patients receiving NNRTI therapy**. No differences were observed between genotypes in regards to serum triglyceride (A) or total cholesterol (B) concentrations. However, carriers of the A allele (closed circles) had significantly decreased HDL-cholesterol values when compared with non-carriers (open circles) at 3, 6, and 12 months after treatment initiation (C). * indicates p < 0.05.

We separately evaluated a subgroup of 113 patients who were undergoing treatment with NNRTIs. Baseline values were also comparable between genotypes, and there were no differences between genotypes with respect to serum triglyceride (Figure 2A) or total cholesterol (Figure 2B) concentrations. However, treatment with NNRTIs significantly increased HDL-cholesterol values in patients with the G/G genotype. This difference with respect to carriers of the A allele was evidenced at three (1.04 ± 0.3 vs. 1.37 ± 0.4 mmol/L; p = 0.021), six (1.03 ± 0.3 vs. 1.30 ± 0.4 mmol/L; p = 0.046), and twelve (1.05 ± 0.3 vs. 1.28 ± 0.4; mmol/L p = 0.05) months (Figure 2C), after adjustment of the data for age, sex, BMI, HCV co-infection status and triglyceride levels.

## Discussion

Our study represents the first report of a strong association between the rs10892151 A allelic variant and the expression, particularly during PI-treatment, of a dyslipidemic phenotype in HIV-infected patients, which includes hypertriglyceridemia and depressed HDL-cholesterol levels.

The high prevalence of lipid disorders among treated HIV-infected patients requires the identification of specific polymorphisms in candidate genes that might predispose to these complications. However, the genetic basis of these metabolic abnormalities remains unclear. Some studies show that variants of apo A-V and C-III, interacting with *APOE *genotypes, are associated with the severity of antiretroviral treatment-induced dyslipidemia. For instance, the most severe dyslipidemic profiles after PI therapy were obtained in those patients carrying the three *APOC3 *variations (i.e., at nucleotides -455, -482 and the *Sst*I site) and a non-ε*3*/ε*3 APOE *genotype [19-21]. Similarly, Guardiola *et al*. [22] indicated that in a cohort of PI treated HIV-infected patients, the -1131C carriers in *APOA5 *gene experienced marked increases in triglyceride levels (up to 80%) during a 3-year follow-up, while no change was recorded in patients carrying the normal -1131T allele. More recent studies [23,24] have associated nucleotide variations in resistin and β2 adrenegic receptor (*ARβ2*) with the occurrence of lipodystrophy. In addition, tumor necrosis factor-α (TNFα) [20,25,26] and nuclear transcription-factor sterol response element-binding protein 1c (SREBP1c) [27,28] have also been shown to affect HIV-induced lipodystrophy, but confirmation is required in long-term prospective studies.

The intriguing results obtained by Pollin *et al*. [5] in the polymorphism rs10892151, which is located 800 kb from the *APOC3 *gene cluster, prompted us to test the presence of a genetic association with lipid outcomes in our patients. In their high-fat feeding intervention study in 809 Old Order Amish individuals, the authors observed that rs10892151 A carriers had lower fasting and post-prandrial serum triglycerides values than non-carriers, and they found a linkage disequilibrium with an *APOC3 *null mutation, which was likely the result of a founder effect. Conversely, we found that this polymorphism in our study has no effect on circulating apo C-III values in either HIV-infected patients or healthy subjects, indicating that the linkage disequilibrium between this two genetic regions could not be universal.

High serum triglyceride concentration and low HDL-cholesterol values were found in our population, irrespective of the treatment they were assigned (although the effect was notably higher in those patients under PI treatment), but these lipid disorders were not related to a differential or abnormal response to circulating apo C-III levels. In addition, we were not able to identify further associations between the polymorphism and other lipid and inflammatory parameters. However, being a carrier of the rs10892151 A variant was a primary determinant of the course of lipid alterations. During follow-up, these patients consistently showed higher serum triglyceride and cholesterol concentrations, as well as lower HDL-cholesterol levels than those carrying the G/G genotype. This effect was particularly evident in those under PI-treatment, although a residual effect in HDL-cholesterol levels was also evident in those under NNRTI-treatment indicating that the HIV-infection itself may have a considerable impact on lipid concentrations for patients with the G/A genotype. However, further studies in larger populations under other chronic inflammatory stimulus are required to confirm the direction of rs10892151 polymorphism effect and to explain the fact that the observed effects in our study were quite different than expected from HIV negative individuals as previously reported [5].

Although the design of our study cannot address the mechanism by which antiretroviral treatment and HIV-infection interact with the polymorphism, it could be argued that antiretroviral treatment may interfere in the functioning of lipolytic enzymes, [29,30] which are regulated by serum apo C-III concentrations, decreasing the catabolism of triglyceride-rich proteins in carriers of the rs10892151 A allele. However it does not explain why antiretroviral treatment and rs10892151 genetic variation have a limited impact on apo C-III levels, probably masked by the concomitant metabolic effects described during HIV infection. Despite the fact that our HIV-infected patients showed higher levels of apo C-III than healthy controls, the described effect was influenced by rs10892151 genotype rather than by serum apo C-III concentration. It is also noteworthy that there is a highly significant gene-nutrient interaction and that dietary manipulation may modulate the effect of polymorphisms on lipid profile [31].

Another possibility is that antiretroviral treatment may not affect just lipoprotein metabolism, but may exacerbate the chronic inflammatory state by the expression of pro-inflammatory molecules such as TNFα or MCP-1, which have a major role in lipid metabolism and, consequently, in cardiovascular disease [14,32-34]. However, this hypothesis is not supported by our findings that the plasma MCP-1 and CRP concentrations were essentially the same in both groups of HIV-infected patients as well as in both genotypes.

The peculiar distribution of alleles seriously limits the reach of our conclusions but the differential response and pharmacogenetic implications among patients with different treatment strongly reinforces the need for replication. According to our data, this should be done in a sample with same ethnic origin and a sample size only achievable in multicenter-multicohort studies. The impact of population stratification in this study is unlikely because in our population sample this has been already assessed empirically by analyzing more than 25 unlinked single-nucleotide polymorphisms (SNPs) in several association studies spanning a range of disease states [35-39] and cases selected under the same circumstances.

We also acknowledge that studies assessing only one polymorphism are less robust than those assessing several polymorphisms associated with a plausible role in the pathogenesis of dyslipidemia. Further studies are needed to assess the possibly coordinated association with other well-documented polymorphisms, particularly those involved in metabolism and in molecular targets of antiretroviral drugs in order to uncover a host-related predisposition towards developing metabolic complications. If successful this may aid for specific, individual design of antiretroviral regimens to diminish the rapid emergence of side effects and the consequent deleterious significance.

## Conclusions

These results suggest that the rs10892151 A allelic variant could be associated in HIV-infected patients with less favorable lipid profile, particularly those treated with PIs. The exact mechanism for this putative effect is unknown but, considering the therapeutic implications, clarification deserves further research.

## Competing interests

The authors declare that they have no competing interests.

## Authors' contributions

JJ and CA-V conceived and designed the study and reviewed critically the manuscript. GA and PP-R performed the statistical analysis and drafted the manuscript. AR, RB-D, ER-G and LF-S collated data or performed various measurements for the study and helped with the analysis and interpretation of the data. JC reviewed critically the manuscript. All authors read and approved the final version of the manuscript.

## Pre-publication history

The pre-publication history for this paper can be accessed here:

http://www.biomedcentral.com/1471-2350/12/120/prepub
